# Hamstring and lower back muscles flexibility as predictor of saddle pressures in young off-road cyclists

**DOI:** 10.3389/fspor.2024.1472550

**Published:** 2024-10-17

**Authors:** Domenico Savio Salvatore Vicari, Antonino Patti, Valerio Giustino, Giacomo Belmonte, Giuseppe Alamia, Marco Gervasi, Eneko Fernández Peña, Antonio Palma, Federico Schena, Antonino Bianco, Ewan Thomas

**Affiliations:** ^1^ Sport and Exercise Sciences Research Unit, Department of Psychology, Educational Science and Human Movement, University of Palermo, Palermo, Italy; ^2^ Department of Neurosciences, Biomedicine and Movement Sciences, University of Verona, Verona, Italy; ^3^ Department of Biomolecular Sciences - Division of Exercise and Health Sciences, University of Urbino Carlo Bo, Urbino, Italy; ^4^ Department of Physical Education and Sport, University of the Basque Country UPV/EHU, Vitoria-Gasteiz, Spain; ^5^ Regional Sports School of Italian National Olympic Committee (CONI) Sicilia, Palermo, Italy

**Keywords:** biomechanics, cycling, bicycle, bike, pelvic tilt, uro-genital pathologies

## Abstract

**Introduction:**

While pedaling, cyclists rest their pelvis on the saddle, generating pressures on it. The pressures generated on the saddle are influenced by several factors. This study aimed to evaluate whether the flexibility of hamstring and lower back muscles could be considered a predictor of pressures in the anterior region (PAR) on the saddle.

**Methods:**

For this study, 15 young off-road Italian cyclists (11m, 4f) aged 13–16 (Italian Federation categories: ES1, ES2, AL1, AL2) were recruited. Each participant was administered the V sit-and-reach (VSR) to measure the hamstring and lower back muscles flexibility. Subsequently, after performing a bike fitting, the saddle pressures during pedaling at three different intensities (100, 140, 180 W), with participants on their own bike installed on specific bike roller, were recorded. The parameters considered for statistical analysis were front pressure (%) and back pressure (%).

**Results:**

The hamstring and lower back muscles flexibility, as result of the VSR test, was a predictor of saddle PAR at 100 W (R^2^ = 0.362, *p* = 0.018), at 140 W (R^2^ = 0.291, *p* = 0.038), and at 180 W (R^2^ = 0.349, *p* = 0.020) of pedaling intensity.

**Conclusion:**

Higher values of the VSR could predict lower values of the pressures exerted in the front region of the saddle. The hamstring and lower back muscles flexibility may be considered a predictor of PAR on the saddle.

## 1 Introduction

Recreational and competitive cycling is constantly expanding and evolving around the world (1, 2). In Italy, in the last decade, off-road cycling has experienced an exponential growth in popularity, thanks also to the contribution of cycling schools sponsored by the Italian Cycling Federation which promote this sport among young people.

Cycling, due to the peculiar position in which athletes must remain for prolonged periods with the trunk leaning forward and maintaining the lumbar flexion to reach the handlebars with the hands, can lead to alterations in the morphology of the spine and pelvis (3). The spine adaptations on the sagittal plane could also be different based on the discipline practiced. In fact, Muyer et al. (2016) found that road cyclists show greater thoracic kyphosis than cross-country cyclists (4). These findings could also depend on the different postures that cyclists adopt on bikes with different geometries. The handlebars of the road bike allow for different grips: low, with greater trunk flexion to be more aerodynamic, and high, with less forward trunk flexion to be more comfortable. The mountain bike, on the other hand, has a handlebar with a single grip and the trunk flexion is influenced only by the bike fitting (5).

In this sport, due to the continuous stresses that occur at the three points of contact between the cyclist and the bike (i.e., saddle, handlebars, and pedals) (6), dysfunctions such as the numbness of the hands, feet, or pubic region can arise (5).

Cross-country, the most popular mountain biking event, is a mass start endurance competition characterized by off-road circuits with continuous climbs and descents on uneven terrains and field trails (7, 8). The higher energy expenditure of off-road cyclists compared to on-road cyclists could also be due to the intense and repeated isometric muscle contractions of upper and lower limbs, necessary to absorb shocks and vibrations caused by uneven terrain. Similarly, athletes require higher levels of energy expenditure to stabilize the bicycle when crossing obstacles and to manage descents and climbs (2, 8). The vibrations caused by the uneven terrain, in addition to causing an increase in energy expenditure, could cause impacts to the cyclist's perineum on the saddle and it would seem that uro-genital pathologies could derive precisely from the repeated microtraumas caused by these continuous impacts (5). As a matter of fact, off-road cyclists usually use bicycles with front or full suspension in order to reduce muscle stress on upper and lower limbs and to absorb shocks on the saddle (7).

While pedaling, cyclists rest their pelvis on the saddle, generating pressures on it. However, abnormal pressures, especially in the anterior region, could cause acute and chronic uro-genital pathologies. The pressures generated on the saddle are influenced by several factors as analyzed in a recent review (5). These factors include the intensity and frequency of pedaling, the cycling discipline, the position of the trunk, and the different anatomical conformation of the pelvis.

As regards the different conformation of the pelvis, related to the athletes’ gender, it has already been extensively described in the scientific literature (9, 10). However, it is also necessary to consider the position of the pelvis in relation to the sagittal plane, which can vary from athlete to another (11). In fact, the pelvis can rotate around the femoral heads, following the bi-coxofemoral axis (12). This anteroposterior rotation represents the pelvic tilt (PT), which is the angle between the vertical line and the line drawn from the center of the femoral heads to the center of the upper sacral endplate (11, 13). When the pelvis rotates backwards (retroversion) the PT increases, while when the pelvis rotates forwards (anteversion) the PT decreases (12, 14). The anteroposterior orientation of the pelvis influences the sagittal curves of the spine. Moreover, reduced flexibility of the hamstring muscles affects pelvic posture and, consequently, the sagittal curves of the spine. Previous studies have shown the effects of reduced hamstring flexibility on PT (15, 16). A study by McEvoy et al. (2007) examined the comparison of mean angles of the anterior PT and the variability of this parameter in elite cyclists and non-cyclists (17). The findings of this study underlined that the mean angles of the anterior PT of elite cyclists were significantly greater and had significantly less variability in non-cyclists when tested in prolonged sitting positions. The study by Muyor et al. (2016), which investigated hamstring flexibility in cross-country cyclists and road cyclists, showed that the latter had greater hamstring flexibility than the former (4). The same research group, in a further study, investigated any correlations between hamstring flexibility and spinal curves and pelvic tilt (18). Their results showed that the hamstring muscles flexibility influence the thoracic and pelvis postures when maximal trunk flexion with knees extended was achieved.

Considering that the hamstring muscles insert at the level of the ischial tuberosity, these can influence the pressures on the saddle (19). That is, these pelvis movements on the sagittal plane (i.e., retroversion and anteversion) could influence the distribution of saddle pressures.

Thus, since saddle pressures can depend on several known and unknown factors, the aim of this study was to evaluate whether the flexibility of hamstring and lower back muscles could be considered a predictor of pressures in the anterior region on the saddle (PAR). Our hypothesis was that PAR on the saddle decreases as the degree of flexibility of the hamstring and lower back muscles increases, which in fact influences the PT.

## 2 Materials and methods

### 2.1 Study design

This is a cross-sectional study in which we evaluated the influence of hamstring and lower back muscles flexibility on saddle pressures in young off-road cyclists of both sexes.

### 2.2 Participants

Fifteen young Italian cyclists (11m, 4f) aged 13 to 16 (Italian Federation categories: ES1, ES2, AL1, AL2) were recruited from local cycling clubs. A member of the Sicilian regional technical research and development committee of the Italian Cycling Federation invited the potential participants who were shown the objectives of the research.

To be eligible for the study, the following inclusion criteria had to be met: (a) be aged between 13 and 16 years; (b) practicing mountain biking for at least 2 years; (c) practicing mountain biking at least 3 h/week. The exclusion criteria were the following: (a) no history of saddle sores; (b) no history of skin irritations in the perineal area, perineal nodules, or perineal numbness; (c) no musculoskeletal injuries in the previous 6 months.

The participation of the cyclists was voluntary and, as minors, the research was also presented to their parents who, by completing and signing the informed consent, agreed to include their children in the study.

The study, in accordance with the principles of the Declaration of Helsinki for the use of people in research, was approved by the Bioethics Committee of the University of Palermo, Italy (n. 132/2023).

### 2.3 Procedure

Each participant was administered the V sit-and-reach (VSR) to measure the hamstring and lower back muscles flexibility and, moreover, the saddle pressures during pedaling at three different intensities (100, 140, 180 W) were recorded.

The test session took place in the laboratory from 3:00 to 6:00 PM during the winter preparation, a period in which there is no competition.

For the VSR test, each participant was seated on the floor with the lower limbs extended, feet spaced 30 cm apart, and the plantar surface of each foot touching a box to keep the ankle joints in a neutral position, forming a V-shape leg position (20, 21). Then, each participant was asked to keep their upper limbs extended, flex their trunk, and reach as far as possible sliding the hands along the floor. Three trials were performed, and the distance reached (cm) in the third trial was measured. Between trials, the participants rested for 1 min.

**Figure 1:**
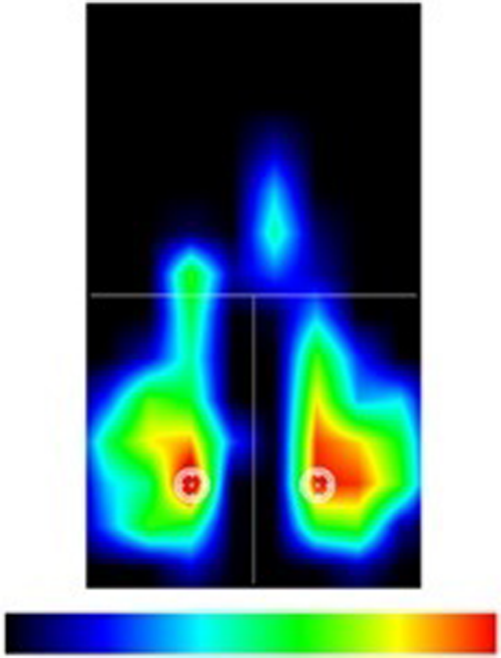
Representation of saddle pressures on a thermographic scale.

Subsequently, the saddle pressures during pedaling at three different intensities (100, 140, 180 W) were recorded. In detail, each participant was evaluated on their own bike installed on specific bike roller (MagneticDays; Foiano della Chiana, Arezzo, Italy) after performing a bike fitting (22–25). The bike fitting was performed with the aim of optimizing the posture and joint functions of each participant (26). Hence, each participant was asked to warm-up for 10-min at a self-selected pedaling cadence and intensity. Then, a flexible mat, composed of resistive sensors capable of recording saddle pressures (W-Saddle Pro, LetSense Group; Castel Maggiore, Bologna, Italy), was placed on a sex-neutral saddle (saddle A, Selle Italia S1; Casella d’Asolo, Treviso, Italia). The resistive sensors of the flexible mat are divided into three regions: pubic region (anterior), left back region, and right back region (Figure 1). Saddle pressures were recorded at 3 different pedaling intensities (100, 140, 180 W) with a pedalling cadence of 90 rpm. Each trial lasted 30 s and a 3-min rest between trials was scheduled. The trials at the different pedaling intensities were carried out in the following block order: (I) 100 W; (II) 140 W; (III) 180 W. The parameters considered for statistical analysis were front pressure (%) and back pressure (%).

### 2.4 Statistical analysis

A descriptive analysis of all variables (means and standard deviations) was performed. Then, the Shapiro-Wilk's test was carried out to check data distribution.

Given the normal distribution of data, a predictive model was constructed using a linear regression analysis with the PAR on the saddle (100, 140, 180 W) as dependent variable and the muscles flexibility scoring as independent variable.

Statistical significance level was set at *p* < 0.05. All statistical analyses were performed using Jamovi software package (version 2.3.28) (27).

## 3 Results

The characteristics of the participants are shown in Table 1.

**Table 1:** Characteristics of the participants.

	Male	Female
*n*	11	4
Age (years)	13.5 ± 1.1	14.5 ± 0.5
Height (cm)	160.3 ± 11.1	162.3 ± 4.11
Weight (kg)	52.6 ± 12.6	53.2 ± 3.58
Body fat (%)	21.36 ± 6.82	25.6 ± 0.79

Values are reported as mean ± SD.

**Figure 2:**
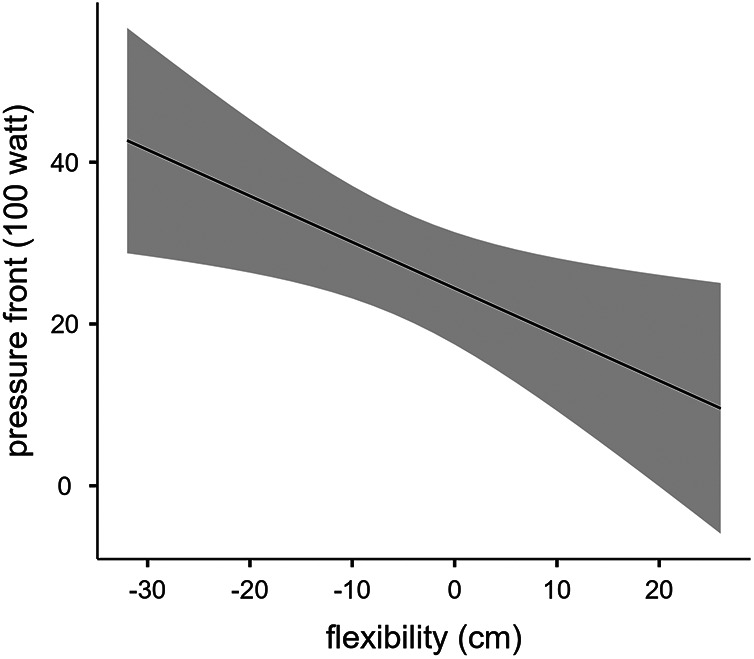
Linear regression between muscles flexibility and saddle PAR at 100 W of pedaling intensity.

**Figure 3:**
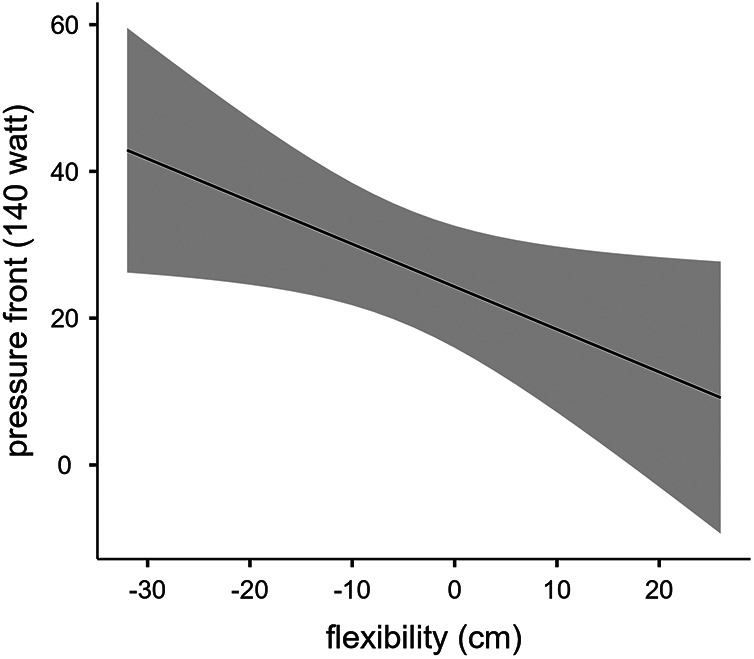
Linear regression between muscles flexibility and saddle PAR at 140 W of pedaling intensity.

**Figure 4:**
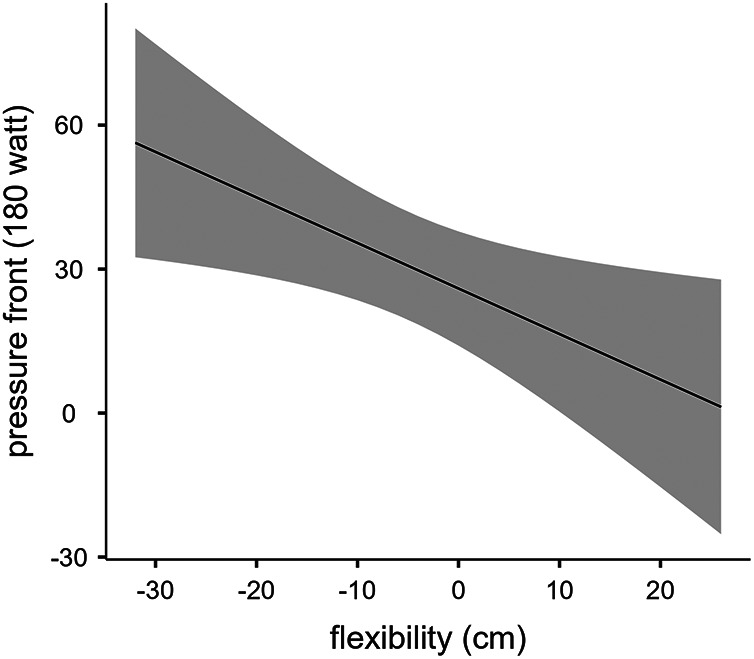
Linear regression between muscles flexibility and saddle PAR at 180 W of pedaling intensity.

Table 2 shows the scores of muscles flexibility of the VSR test, and the values of saddle PAR recorded at the 3 different pedaling intensities (100, 140, 180 W). The linear regression analysis showed that muscles flexibility was predictive of saddle PAR at 100 W (R^2^ = 0.362, *p* = 0.018), at 140 W (R^2^ = 0.291, *p* = 0.038), and at 180 W (R^2^ = 0.349, *p* = 0.020) of pedaling intensity, as shown in Figures 2–4 respectively.

**Table 2:** Descriptive analysis of the performances of muscle flexibility and front pressure on the saddle.

VSR test (cm)	-4.93 ± 15.0
PAR (%) at 100 W	27.2 ± 14.2
PAR (%) at 140 W	27.2 ± 16.2
PAR (%) at 180 W	30.6 ± 24.1

Values are reported as mean ± SD. VSR, V sit-and-reach test; PAR, pressures in the anterior region of the saddle; W, watt.

## 4 Discussion

The aim of this study was to evaluate whether the flexibility of hamstring and lower back muscles could be considered a predictor of PAR on the saddle. Our hypothesis was a decrease in PAR on the saddle as the degree of flexibility of hamstring and lower back muscles increases. Our results confirmed our hypothesis, in fact, as the level of muscles flexibility increases, lower pressures were recorded in the anterior region of the saddle.

Studies on biomechanics are of crucial importance for preventing injuries (28), and due to the nature of cross-country races, that take place on uneven terrain, vibrations and impacts on the saddle could lead to repeated microtraumas with consequent uro-genital pathologies. Knowing the attributes related to saddle pressures could prevent the onset of these pathologies. Indeed, the practice of one discipline rather than another can lead, over time, to different adaptations in the cyclist's posture (4).

A recent study analysed the effects of practicing road and cross-country discipline on sagittal spine curves, on pelvic tilt, and trunk tilt showing that road cyclists have a greater thoracic kyphosis and a greater forward torso tilt than cross-country cyclists (4). These adaptations could be due to the different posture adopted. In fact, road cyclists have a greater saddle-handlebar height difference than cross-country cyclists. The same research group investigated the difference of road and cross-country cyclists on the extensibility of the hamstring muscles. Authors detected that road cyclists have a greater hamstring extensibility than cross-country cyclists (4). These findings could be explained by the different trunk position in these disciplines in which a greater trunk flexion is required in road cyclists. Previous studies have confirmed the relationship between the degree of muscle flexibility and pelvic position in the sagittal plane. A study by Muyor et al. (2012) reported that the pelvis rotates forward after hamstring stretching (16). Similarly, Feland et al. (2001) confirmed that pelvic mobility in the sagittal plane increases after hamstring stretching in elderly people (29). Indeed, previous research groups showed that a greater hamstring muscle flexibility allow to reach a higher anterior PT than cyclists with a lower hamstring muscle flexibility (4, 30).

These studies are in line with our results, underlining that the pelvic position plays a key role in the distribution of pressures on the saddle. The present study showed that off-road cyclists who had a greater flexibility of hamstring and lower back muscles reported a lower saddle pressure in the anterior region. These findings seem to be in contrast with most of the existing literature. Moreover, in cross-country cycling, to promote forward movement on the saddle especially on steep climbs and to avoid lower back pain, the saddle is positioned with the nose tilted down few degrees, this would allow the pelvis to rotate forward. The latter, together with the greater capacity to extend the hamstring muscles, would promote the anterior PT with a greater possibility of PAR on the saddle. However, our interpretation for the results found are in line with the abovementioned studies as these could be explained by the fact that these cyclists are better able to manage PT on the saddle compared to those with less muscles flexibility.

A recent review describes the factors that influence saddle pressures in order to prevent the related pathologies (5). As a matter of fact, some studies in the scientific literature have analysed the relationship between the use of different saddles and PT. The study by Bressel et al. (2003) examined the PT, trunk angle, and electromyography in 20 female cyclists (10 experienced and 10 novice) while pedaling on stationary cycle ergometers on three different saddles (without cutout, with partial cutout, with a complete cutout) (31). The authors’ results showed that saddles with cutout in the front promote a forward PT. Promoting a forward PT and trunk may help distribute a greater percentage of body weight over the handlebars, reducing the load on the saddle and spine (32). Indeed, it seems that the anterior tilt of the pelvis and trunk can reduce the incidence of low back pain (33). However, there are further studies that agree with our findings confirming that a forward PT increases the pressure exerted on the anterior perineum (31). The study by McEvoy et al. (2007) found greater anterior PT in elite cyclists than non-cyclists when sitting on the floor with knees extended (17). This difference could be related to greater hamstring flexibility in cyclists. Furthermore, authors stated that an increased anterior PT facilitates the aerodynamics of competitive road cyclists. Therefore, greater flexibility of the hamstring and lower back muscles could increase the anterior PT and, consequently, facilitate the aerodynamic position of the cyclists.

Based on our results, the flexibility of the hamstring and lower back muscles could be a factor that can influence the distribution of pressures in the saddle. It should be noted that excessive pressure on the anterior region of the saddle can lead to hypoxia of the nerve, especially if this pressure is prolonged over time. Previous studies showed that an excessive pressure in the anterior region of the saddle is detrimental to the erectile tissues (9, 34). For this reason, reducing the compressive load on soft tissues is the main objective of bike fitting in order to prevent these pathologies.

## 5 Conclusions

The flexibility of hamstring and lower back muscles could be considered a predictor of pressure on the anterior region of the saddle. In particular, lower values from the VSR test could be indicative of higher values of PAR on the saddle. Coaches and cyclists should consider assessing the flexibility of hamstrings and lower back muscles to manage PAR. This assessment could be useful to optimise cyclist's position and, eventually, to plan a stretching training program.

## Data Availability

The raw data supporting the conclusions of this article will be made available by the authors, without undue reservation.
